# Professional Reasoning in Dietary Hyperkalaemia Management in Chronic Kidney Disease: Development of the PRIME‐K Model

**DOI:** 10.1111/jhn.70314

**Published:** 2026-07-17

**Authors:** Andrew Morris, Kelly Lambert

**Affiliations:** ^1^ Research Centre for Healthcare and Community Transformation Coventry University Coventry UK; ^2^ Dietetics Worcestershire Acute Hospitals NHS Trust, Charles Hastings Way Worcester UK; ^3^ School of Medical, Indigenous and Health Sciences University of Wollongong Wollongong Australia

**Keywords:** clinical decision‐making, diet therapy, dietitians, hyperkalemia, renal insufficiency

## Abstract

**Background:**

Dietary management of hyperkalaemia in chronic kidney disease has traditionally focused on potassium restriction. However, contemporary renal nutrition practice increasingly requires dietitians to balance biochemical safety with nutritional adequacy, dietary quality, treatment burden, and patient wellbeing. Limited empirical evidence has examined how renal dietitians reason through these competing priorities in routine practice.

**Objective:**

To explore how renal dietitians approach dietary hyperkalaemia management in chronic kidney disease and to examine the professional reasoning underpinning dietary decision‐making.

**Methods:**

This qualitative study integrated free‐text responses from an international online survey of renal dietitians with semi‐structured interviews. Survey free‐text responses provided breadth across practice settings, while interviews enabled more detailed exploration of clinical reasoning. Data from 203 survey respondents and eight interview participants were analysed consequently using reflexive thematic analysis to examine how dietitians prioritised, sequenced, and adapted dietary interventions.

**Results:**

Dietitians described dietary hyperkalaemia management as a staged but flexible process of professional reasoning. Four interrelated themes were identified: consideration of non‐dietary contributors before dietary restriction; prioritisation of potassium additives and highly processed foods; preservation of nutritional adequacy as a central boundary condition; and selective restriction of potassium‐rich whole foods only when clinically indicated. Dietary restriction was not described as an automatic response to elevated serum potassium, but as one possible component of a broader reasoning process shaped by biochemical trends, nutritional status, clinical urgency, patient preferences, and psychosocial context. These findings informed development of the Prioritisation and Reasoning in the Management of Elevated Potassium in Chronic Kidney Disease model, known as PRIME‐K.

**Conclusions:**

Renal dietitians reported using flexible professional judgement to manage hyperkalaemia while minimising unnecessary dietary burden and protecting nutritional adequacy. The PRIME‐K model offers a practice‐informed representation of professional reasoning in dietary hyperkalaemia management and may support dietetic education, reflective practice, and the development of more context‐sensitive approaches to dietary care in chronic kidney disease.

## Summary

- Renal dietitians described dietary hyperkalaemia management in chronic kidney disease as a staged but flexible process, with non‐dietary contributors commonly considered before dietary modification.
- Lower‐burden strategies, including reducing potassium‐containing additives, salt substitutes, and highly processed foods, were often prioritised before restriction of potassium‐rich whole foods.
- Professional reasoning was shaped by the need to balance potassium control with nutritional adequacy, dietary quality, patient preferences, and clinical context, resulting in individualised rather than prescriptive dietary advice.

## 1 Introduction

Hyperkalaemia is a common and potentially serious complication of chronic kidney disease and kidney replacement therapy, associated with cardiac arrhythmias, hospitalisation, and increased mortality [1, 2]. Dietary management has historically focused on restricting potassium‐rich foods, often implemented through lists of foods to avoid [3, 4]. While this approach may be pragmatic in some clinical contexts, it is increasingly recognised as potentially inconsistent with contemporary, patient‐centred renal nutrition care [5].

Serum potassium concentration is influenced by a complex interplay of dietary, physiological, pharmacological, and treatment‐related factors [2]. Constipation, metabolic acidosis, glycaemic control, medication use, dialysis adequacy, catabolic states, and pre‐analytical sampling issues may all contribute to hyperkalaemia independently of dietary intake [2]. Consequently, elevated serum potassium does not necessarily indicate excessive dietary potassium intake. Dietary restriction applied without adequate assessment may therefore risk misattributing causality and placing unnecessary burden on patients, particularly those already vulnerable to poor nutritional status, frailty, or reduced quality of life [2, 5].

Contemporary nutritional guidance in chronic kidney disease increasingly emphasises dietary quality, fibre intake, plant‐based foods, and cardiometabolic health [6, 7, 8]. Emerging evidence also suggests that potassium derived from whole, minimally processed plant foods may differ in bioavailability from potassium salts used as additives in processed foods [9]. Broad restriction of fruits, vegetables, legumes, and wholegrains may therefore conflict with wider nutritional priorities, including prevention of protein–energy wasting, constipation, cardiovascular disease, and poor overall diet quality [7].

Dietary hyperkalaemia management therefore requires renal dietitians to balance competing priorities, including biochemical safety, nutritional adequacy, dietary quality, patient preferences, and treatment burden [5, 8]. This work involves more than the application of dietary guidelines or nutrient thresholds alone. It requires professional judgement to determine whether dietary intervention is warranted, which factors should be addressed first, and what form dietary advice should take within the context of a patient's clinical and psychosocial circumstances.

Although individual components of contemporary hyperkalaemia management have been described in guidance [6, 8] and previous literature [2] such as dietary counselling, and selective use of binders; less is known about how renal dietitians prioritise, sequence, and adapt these components in routine practice. Understanding this reasoning is important because dietary hyperkalaemia management is not simply a question of what foods contain potassium, but of how dietitians decide when, how, and to what extent dietary change is appropriate.

Therefore, this study aimed to explore how renal dietitians approach dietary hyperkalaemia management in chronic kidney disease, with particular focus on the professional reasoning underpinning dietary decision‐making in routine practice. A secondary aim was to synthesise these findings into a conceptual model describing how renal dietitians prioritise and adapt dietary interventions when managing elevated potassium in chronic kidney disease.

## 2 Methods

### 2.1 Study Design

This study used a qualitatively driven, sequential multi‐source design embedded within a wider international online survey. This paper presents a secondary, in‐depth qualitative analysis of interview data collected during the original study. The original study was designed to provide a broad account of dietitians' experiences and practices in the dietary management of hyperkalaemia, and a summary of the initial themes has been reported elsewhere. However, during the initial analysis and subsequent interpretation of the findings, it became apparent that participants' accounts contained a more nuanced layer of professional reasoning, including how dietitians interpret biochemical, clinical and dietary information; manage uncertainty; prioritise referrals; and balance potassium reduction with nutritional adequacy, patient preference and wider clinical risk.

The present analysis was therefore undertaken to examine this dimension of the data in greater depth. Rather than repeating the original thematic findings, this secondary analysis addresses a distinct research question focused specifically on professional reasoning and decision‐making in practice. This required a more interpretive and fine‐grained analysis of the existing dataset, with attention to the judgements, assumptions, contextual factors and tacit knowledge underpinning dietitians' management of hyperkalaemia.

Survey free‐text responses were analysed initially to identify preliminary patterns in renal dietitians' professional reasoning, inform purposive sampling for follow‐up interviews, and guide development of the semi‐structured interview topic guide. Interviews were subsequently undertaken to explore these emerging patterns in greater depth.

This design was selected because survey free‐text responses provided breadth across renal dietetic settings, geographical contexts, and areas of practice, whereas interviews enabled in‐depth exploration of how dietitians reasoned through specific clinical decisions. This study examined how renal dietitians described decision‐making in response to elevated serum potassium, including how they considered non‐dietary contributors to hyperkalaemia, sequenced dietary strategies, balanced nutritional adequacy with biochemical management, and judged the proportionality of dietary potassium restriction.

The qualitative components were reported with reference to established qualitative reporting guidance, including principles of transparency in sampling, data collection, reflexivity, and analytic interpretation [10]. The interview component is reported in line with the COREQ. 32‐item checklist [11].

### 2.2 Participants and Recruitment

Dietitians with current or recent experience in adult renal services were recruited internationally between July and December 2024 via professional organisations, renal nutrition networks, conference dissemination, social media posts, and professional contacts. The online survey used convenience sampling.

Respondents who wished to be interviewed provided contact details separately via email to the Principal Investigator (PI) AM. Participants for the second set of qualitative interviews were selected from individuals who had completed the survey and indicated willingness to participate in follow‐up qualitative research. A purposive sampling approach was used to identify participants who could provide additional depth and variation in perspectives relevant to the survey findings. Where possible, selection considered professional and practice‐related characteristics, including country or region of practice, clinical role, years of experience, and practice setting. Eligible participants were contacted by email with further information about the interview component, and those who agreed provided informed consent prior to interview. The purpose of this second qualitative phase was to elaborate and contextualise survey‐derived findings rather than to generate a statistically representative sample.

### 2.3 Original Data Collection

An online survey was developed to explore dietary management of hyperkalaemia in chronic kidney disease and distributed online via Jisc Online Surveys for 6 months in 2024. The survey included demographic questions, structured items relating to clinical practice, and free‐text questions examining professional reasoning, decision‐making, perceived challenges, and factors influencing dietary advice.

Free‐text questions invited respondents to describe how they approached dietary management when serum potassium was elevated, what factors they considered before recommending dietary restriction, how they prioritised different dietary strategies, and how they balanced potassium management with broader nutritional goals.

Semi‐structured interviews were conducted with a purposive subsample of survey respondents. The purpose was not to achieve independent thematic saturation, but to deepen, contextualise, and refine interpretation of findings generated from the larger survey dataset. Interviews explored professional reasoning in greater depth, including how dietitians interpreted biochemical results, assessed possible contributors to hyperkalaemia, determined whether dietary change was required, and adapted advice to patients' nutritional status, preferences, comorbidities, and social circumstances.

The interview guide [Supplement S1] was informed by relevant literature, clinical knowledge of renal dietetic practice, and preliminary review of survey free‐text responses. Interviews were conducted remotely by [AM and KL], audio‐recorded with consent via Microsoft Office Teams, and transcribed verbatim using Microsoft Office Teams. Transcripts were reviewed for accuracy against the original recording by AM.

### 2.4 Data Analysis

Data were analysed using reflexive thematic analysis, following the approach described by Braun and Clarke [12, 13]. This approach was selected because the study aimed to examine patterns of meaning in dietitians' accounts of professional reasoning, rather than to quantify predefined categories of practice or establish coding reliability. Reflexive thematic analysis was therefore appropriate for exploring how dietitians described prioritising, sequencing, and adapting dietary interventions for hyperkalaemia across varied clinical contexts. In line with reflexive thematic analysis, the interview data were analysed iteratively and interpretively, with attention to how participants' accounts elaborated, complicated, or refined survey‐derived themes rather than simply confirming pre‐existing categories.

Analysis occurred in two connected stages. Microsoft Excel was used for all coding and theme development. First, survey free‐text responses were analysed to identify broad patterns in dietitians' reported approaches to hyperkalaemia management and to develop an initial understanding of the professional reasoning underpinning these approaches. The survey data provided breadth across a large international sample and helped identify recurring decision points, perceived causes of hyperkalaemia, intervention strategies, thresholds for dietary restriction, and contextual factors influencing practice. This preliminary analysis informed the development of the interview topic guide and helped identify areas requiring deeper exploration.

Participants who had completed the survey and indicated willingness to take part in a follow‐up interview were then purposively selected for in‐depth interviews. Selection was informed by the emerging survey analysis and aimed to explore professional reasoning themes identified in the larger dataset in greater depth. Interview participants were selected to capture variation in accounts where possible, including differences in clinical setting, service context, geographical location, patient population, and reported approaches to dietary potassium management. The interviews therefore enabled more detailed exploration of how dietitians reasoned through specific clinical decisions, including how they weighed biochemical urgency, nutritional risk, patient preferences, comorbidities, medication use, and service constraints.

Following completion of the interviews, survey free‐text responses and interview transcripts were analysed as a connected qualitative dataset, while retaining awareness of the different nature and function of each data source. Survey free‐text responses were used to establish breadth and identify recurring patterns across the wider sample, whereas interview transcripts provided richer, more detailed accounts of how dietitians explained, justified, and adapted their clinical decisions. The source of each extract was retained throughout analysis so that emerging interpretations could be examined across both survey and interview data.

AM coded the survey free‐text responses first. Initial coding was primarily inductive, with codes developed from participants' accounts rather than imposed from a pre‐existing framework. However, the analysis was sensitised by the study aim of examining professional reasoning in dietetic management of hyperkalaemia. Coding therefore focused not only on what dietary interventions participants reported, but also on how they described prioritising, sequencing, adapting, or avoiding these interventions. Early codes captured decision points, perceived causes of elevated potassium, dietary strategies, thresholds for restriction, contextual influences, patient‐related factors, nutritional concerns, and explanations for dietary choices.

After the interviews had been completed and transcribed, AM coded the interview transcripts, using the preliminary survey analysis as a sensitising foundation while remaining open to new concepts, contradictions, and refinements. Interview coding explored how participants elaborated on, complicated, or challenged themes that had emerged from the survey data. Attention was paid to the reasoning processes through which dietitians balanced potassium reduction with nutritional adequacy, quality of life, patient burden, clinical urgency, and the practical realities of service delivery. Co‐coding was not used to establish inter‐rater reliability, as this would be inconsistent with reflexive thematic analysis. Instead, it was used to challenge assumptions, explore alternative interpretations, and refine the developing analysis. Coding decisions, emerging interpretations, and areas of uncertainty were discussed between AM and KL, with disagreements treated as opportunities for analytic refinement rather than as errors requiring consensus scoring.

Codes were iteratively refined through repeated engagement with both survey and interview data. Related codes were grouped into candidate themes representing shared patterns in how dietitians described managing hyperkalaemia. Candidate themes were developed through memo‐writing, comparison across data sources, iterative discussion, and repeated checking against the full dataset. Themes were reviewed against coded extracts and the wider dataset to ensure that they captured both common reasoning processes and important areas of variation. This included checking whether themes identified in the survey data were elaborated, modified, or challenged in interview accounts.

Attention was paid to convergence and divergence across participants' accounts. Where differences in practice were identified, these were examined in relation to clinical setting, service context, perceived urgency, patient complexity, stage of kidney disease, dialysis status, nutritional risk, and availability of multidisciplinary support. The analysis therefore sought not only to identify common dietary strategies, but to understand the reasoning through which these strategies were prioritised, sequenced, adapted, or withheld.

Reflexivity was maintained throughout the analytic process. The research team recognised that their professional and disciplinary backgrounds could shape the interpretation of participants' accounts, particularly in relation to assumptions about dietetic practice, risk management, and evidence‐based dietary care. Reflexive memoing and team discussions were used to make these assumptions explicit and to consider how they influenced coding, theme development, and interpretation. Analytic decisions were documented throughout, including changes to code labels, theme boundaries, and the developing relationships between themes.

### 2.5 Development of the PRIME‐K Model

The PRIME‐K model was then developed by examining how the final themes related to one another within participants' accounts of practice. The model was not imposed at the outset but was generated from the integrated analysis of survey and interview data as an interpretive representation of how dietitians described assessing, prioritising, and adapting dietary management of hyperkalaemia. The final themes and model were checked against the full dataset to ensure they reflected both recurring patterns from the wider survey sample and the more detailed reasoning captured through interviews.

The PRIME‐K model was designed to represent a staged but flexible process rather than a prescriptive algorithm. It reflects how dietitians described moving between consideration of non‐dietary contributors, lower‐burden dietary modifications, protection of nutritional adequacy, and selective restriction of potassium‐rich whole foods when clinically indicated. The model also incorporates the iterative nature of practice, recognising that clinical urgency, biochemical trends, nutritional risk, patient preferences, and service constraints may alter the order or intensity of intervention.

### 2.6 Reflexivity and Rigour

At the time of data collection, AM had 17 years' experience as a renal dietitian and working as a clinical academic. KL was an academic with 30 years of renal clinical practice. The analysis was informed by the authors' experience and expertise in renal nutrition and clinical dietetic practice. This professional background supported interpretation of clinically nuanced accounts but also required reflexive attention to assumptions about appropriate hyperkalaemia management. Reflexive discussion was used throughout analysis to consider how the authors' clinical knowledge, professional roles, and expectations may have shaped coding, theme development, and interpretation.

Rigour was supported through repeated reading of the data, systematic coding, discussion of developing themes between authors, comparison of survey and interview accounts, and active consideration of variation and exceptions within the dataset. Quotations were selected to illustrate both shared patterns and clinically relevant differences in reasoning.

No prior relationship existed with most participants before recruitment. However, participants may have recognised the investigators through professional renal dietetic networks, the study information sheet clarified that the study sought to understand a range of routine reasoning practices rather than endorse a preferred dietary approach.

### 2.7 Ethical Considerations

Ethical approval for the original study was granted by Coventry Univesity (project reference number P178765). The current analysis used the existing anonymised dataset and remained within the scope of participants' original consent. Data were handled in accordance with the original approval and data management procedures. All participants provided informed consent prior to participation. Survey respondents were informed that participation was voluntary and that they could choose whether to express interest in a follow‐up interview. Interview participants provided additional consent for audio‐recording and use of anonymised quotations. Data were anonymised prior to analysis, and identifying details were removed from participant quotations.

To support psychological safety, participants were reminded that participation was voluntary, that they could decline to answer any question or withdraw, and that data would be de‐identified. The interviewer encouraged respectful discussion and made clear that there were no expected or preferred responses. Potential power dynamics were considered, including differences in professional seniority, clinical experience, and practice setting, which may have influenced participants' willingness to share dissenting or critical views.

## 3 Results

### 3.1 Participant Characteristics

A total of 203 renal dietitians from 14 countries completed the online survey. Participants were predominantly female (94%), with an estimated mean age of 42.2 years (SD 10.9) and an estimated average of 12.6 years (SD 9.1) of experience in renal dietetics across multiple settings and all stages of CKD (Table 1).

**Table 1:** Demographics of participants who took part in the online survey.

Characteristic	*n* (%)
Country/Region	
United Kingdom	102 (50.2%)
Australia/New Zealand	36 (17.7%)
North America	28 (13.8%)
Europe	24 (11.8%)
Other (Asia, Africa, Middle East)	13 (6.4%)
Years in renal dietetic practice	
Less than 5 years	41 (20.2%)
5–10 years	48 (23.6%)
11–20 years	61 (30.0%)
21–30 years	38 (18.7%)
More than 30 years	15 (7.4%)
Primary work setting	
Outpatient clinic	92 (45.3%)
Inpatient acute care	61 (30.0%)
Dialysis unit	30 (14.8%)
Community/home visits	12 (5.9%)

Eight participants from three countries took part in follow‐up semi‐structured interviews. Fourteen participants had initially expressed an interest in taking part in the interviews however four did not respond to follow‐up emails from the PI, one withdrew with no explanation, and one withdrew for health reasons. Interview participants were all female, with an estimated mean age of 42.1 years (SD 8.7) and an estimated mean of 14.9 years (SD 7.2) of renal dietetic experience across three countries: Australia, Canada and the United Kingdom, and different settings and CKD stages (Table 2). The mean interview time was 72 min. No interviews were repeated.

**Table 2:** Demographics of participants who took part in the semi‐structured interviews.

Participant	Country	Gender	Age group	Highest degree	Years of renal dietetic experience	Role	CKD group	Non‐medical prescribing qualification
P1	Canada	Female	40–49 years	Batchelor	11–20 years	Inpatient, outpatient, pre‐dialysis, conservative care, early CKD, peritoneal dialysis	Stage 3–5 (no dialysis or transplantation/PD)	No
P2	Australia	Female	40–49 years	Batchelor	11–20 years	Outpatient, pre‐dialysis, early CKD, Haemodialysis unit, peritoneal dialysis unit	Stage 2–5 (HD and PD)	No
P3	Canada	Female	40–49 years	Batchelor	11–20 years	Outpatients	Stage 1–3	No
P4	Australia	Female	30–39 years	Batchelor	11–20 years	Inpatient, outpatient, pre‐dialysis, conservative kidney management, haemodialysis unit, transplant unit, peritoneal dialysis unit.	Stages 3–5 (HD, PD, TX, Conservative care)	No
P5	Australia	Female	50–59 years	Masters	21–30 years	Outpatient, pre‐dialysis, early CKD, Haemodialysis unit, peritoneal dialysis unit	Stages 2–5 (HD, PD, TX, conservative care)	No
P6	Canada	Female	21–29 years	Batchelor	0–2 years	Outpatient, transplant unit	Stage 5 transplant	No
P7	UK	Female	40–49 years	Masters	11–20 years	Inpatient, outpatient, pre‐dialysis, conservative care, haemodialysis, catering/food service department, leadership/management role	Stage 4–5 (HD and conservative care)	Yes
P8	Canada	Female	40–49 years	Batchelor	11–20 years	Outpatient	Stage 1–3	No

### 3.2 Overview of Findings

Four interrelated themes were identified describing how renal dietitians approach dietary hyperkalaemia management in chronic kidney disease. Across accounts, dietary management was characterised as a staged but flexible process of professional reasoning, in which interventions were prioritised according to perceived clinical necessity, reversibility, patient context, and potential impact on nutritional status.

The four themes were: consideration of non‐dietary contributors prior to dietary restriction; prioritisation of potassium additives and highly processed foods; preservation of nutritional adequacy and dietary quality; and selective restriction of potassium‐rich whole foods when clinically indicated. Although a broadly shared reasoning structure was evident, participants described variability in the sequencing and intensity of intervention depending on clinical urgency, patient complexity, patient preferences, and service context.

Table 3 illustrates the analytic development of the themes from initial codes and their contribution to the PRIME‐K model.

**Table 3:** Development of themes and mapping to the PRIME‐K model.

Final theme	Analytic category	Illustrative codes	Exemplar quotation	Contribution to PRIME‐K model
1. Consideration of non‐dietary contributors prior to dietary restriction	Clinical attribution before dietary intervention	Medication review; bowel function; constipation; metabolic acidosis; glycaemic control; dialysis adequacy; potassium trends; laboratory or sampling issues	“Start with other causes/contributors of high potassium, then with diet.”	P — Prioritise clinical attribution before dietary restriction
	Whole‐case review before restriction	Review the case as a whole; avoid assuming diet is causal; address reversible contributors first	“Prior to dietary intervention it is important to review the case. If hyperkalaemia is caused by non‐dietary factors, address those first.”	P — Prioritise reversible non‐dietary contributors
	Medical optimisation and multidisciplinary coordination	Medication changes; sodium bicarbonate supplementation; diabetes management; constipation management; regular blood monitoring	“Regular blood tests and medical management as appropriate such as change of medications, sodium bicarbonate supplementation, diabetes management, constipation management etc.”	P/R — Prioritise and reason across multidisciplinary contributors
2. Prioritisation of potassium additives and highly processed foods	Targeting high‐bioavailability potassium sources	Potassium additives; potassium chloride; salt substitutes; processed foods; canned foods; additive‐containing foods	“Target potassium additives first as they are quick wins because they're more bioavailable”	R — Reduce lower‐value, high‐bioavailability potassium sources first
	Lower‐burden dietary modification	Processed foods first; keep advice simple; identify modifiable sources; reduce hidden potassium exposure	“I try to focus on avoiding processed foods first and keep it simple.”	R — Reduce potassium burden while minimising dietary impact
	Label reading and feasibility of change	Ingredient lists; additive detection; patient education; food access; food insecurity; reliance on processed foods	“Read ingredients for processed foods where potassium may appear and avoid potassium additives where possible.”	I — Individualise advice according to feasibility, access, and context
3. Preservation of nutritional adequacy and dietary quality	Avoidance of unnecessary restriction	Least restrictive approach; avoid default fruit and vegetable restriction; protect dietary variety; avoid blanket food bans	“Overall, I try to be the least restrictive as possible, and I only restrict plant‐based foods as the last resort.”	M — Maintain nutritional adequacy and dietary quality
	Nutritional adequacy as a limiting condition	Protect protein and energy intake; preserve fibre; maintain diet quality; consider frailty and malnutrition risk	“I prefer to identify a few specific high potassium sources, the removal of which won't significantly affect their overall dietary nutritional quality.”	M — Maintain adequacy as a boundary condition on restriction
	Whole‐diet framing	Plant‐forward advice; Mediterranean‐style dietary patterns; fibre intake; gut health; constipation prevention	“Plenty of whole grains and fluids to avoid constipation and maintain healthy gut bacteria.”	M — Maintain whole‐diet quality rather than focusing on potassium alone
4. Selective restriction of potassium‐rich whole foods when clinically indicated	Graduated escalation of dietary restriction	Stepwise approach; additives before whole foods; concentrated sources before broad restriction; persistent hyperkalaemia	“If other causes for high potassium have been ruled out, I will recommend restriction in a stepwise approach.”	E — Escalate restriction only when clinically indicated
	Portion, frequency, and preparation rather than elimination	Scale‐back approach; portion reduction; frequency modification; food swaps; boiling and draining potatoes; cooking methods	“We talk about portion size/frequency of food/fluid items… often you do not need to completely remove potassium‐rich sources.”	E — Escalate proportionately through modification before exclusion
	Targeting concentrated potassium sources	Juices; smoothies; soups; tomato sauces; large portions; habitual high‐potassium combinations	“Avoid concentrated sources of potassium such as juice, smoothies, tomato sauces/soups.”	E — Escalate selectively by targeting concentrated sources
	Escalation beyond diet where needed	Potassium binders; medical management when diet is insufficient; avoid excessive dietary restriction	“If potassium remains high, I recommend potassium binders.”	K — Keep reviewing potassium response and consider non‐dietary escalation when diet alone is insufficient

*Note:* This table illustrates the analytic progression from initial codes to final themes and their contribution to the PRIME‐K model. It is not intended to represent a linear coding process; rather, it shows how recurring patterns across survey free‐text responses and interview transcripts were synthesised into themes and used to inform the conceptual model. PRIME‐K: P, prioritise clinical attribution before dietary restriction; R, reduce lower‐value, high‐bioavailability potassium sources first; I, individualise advice according to patient context and feasibility; M, maintain nutritional adequacy and dietary quality; E, escalate dietary restriction selectively and proportionately; K, keep monitoring potassium response and revising the management plan.

### 3.3 Theme 1: Consideration of Non‐Dietary Contributors Prior to Dietary Restriction

This theme describes how dietary modification was typically not reported as the first response to hyperkalaemia. Instead, participants described an initial phase of attribution, in which they considered whether elevated serum potassium was likely to be dietary, non‐dietary, or multifactorial. Non‐dietary contributors were considered and, where possible, addressed before dietary restriction was intensified.

This reasoning reflected a desire to avoid premature dietary modification and to ensure that reversible medical contributors were not overlooked. Commonly identified factors included bowel function, medication effects, acid–base balance, glycaemic control, dialysis adequacy, and laboratory or sampling‐related artefacts.

I would never start restricting food without first addressing other factors—bowel habits, bicarbonate, meds. That's just unsafe.

Dietitian, UK [Online free text]

Too often potassium gets blamed on diet when there are multiple other contributors that haven't been addressed.

Dietitian, Australia [interview]

Participants also described potassium management as requiring coordination with wider medical management, particularly where medication changes, sodium bicarbonate supplementation, diabetes management, constipation treatment, or monitoring of biochemical trends were relevant.

Regular blood tests and medical management as appropriate such as change of medications, sodium bicarbonate supplementation, diabetes management, constipation management etc.

Dietitian, Netherlands [Online free text]

Participants described this process in both structured and intuitive ways. For some, it resembled a mental checklist, while for others it was embedded in broader clinical judgement.

It's not a tick‐box, but there is a mental sequence…you rule things out before you blame diet.

Dietitian, Canada [Online free text]

However, this sequencing was not universal. In acute care contexts, participants described simultaneous rather than sequential reasoning, where dietary advice was delivered alongside medical management.

In acute admissions you're doing everything at once…you don't have time to work through it step by step.

Dietitian, North America [Online free text]

Participants also identified dialysis adequacy as an important non‐dietary consideration, particularly where measures such as Kt/V or urea clearance were below recommended levels. In these accounts, persistent hyperkalaemia was interpreted as potentially reflecting inadequate potassium removal rather than excessive dietary intake alone.

I would also check dialysis adequacy. Kt/V, urea clearance, because if they're not clearing potassium then that's your issue, not diet. They get referred for a low potassium diet and when you do the diet history its low anyway because they're not feeling great.

Dietitian, UK [interview]

### 3.4 Theme 2: Prioritisation of Potassium Additives and Highly Processed Foods

This theme captures how dietary intervention, when required, was often initially directed towards non‐whole food sources of potassium, particularly potassium‐containing additives, highly processed foods, and salt substitutes. Participants described these sources as important early targets because they were perceived to contribute to potassium exposure while offering fewer nutritional benefits than whole plant foods.

Dietitians frequently framed potassium additives as an early target for intervention because this approach was perceived as lower burden than restricting whole plant foods.

Target potassium additives first as they're more absorbable than whole foods, according to the food science research.

Dietitian, South America [Online free text]

This approach was commonly linked to highly processed foods, salt substitutes, and other sources of hidden or additive potassium.

First line of focus will be processed high‐[potassium] sources and [potassium] additives.

Dietitian, North America [Online free text]

Practical strategies included supporting patients to identify potassium‐containing additives through label reading, discussing the use of potassium chloride salt substitutes, and identifying processed or convenience foods that could be modified or substituted. Label reading was described as a practical mechanism through which patients could identify hidden or additive sources of potassium, although participants often framed this advice as something to be adapted to the patient's literacy, resources, and food access.

Read ingredients for processed foods where potassium may appear… and avoid potassium additives where possible, and where appetite is good. You also need to consider peoples families that they need to feed as well… you need to be aware of the bigger picture and make sure you cover all bases in your assessment.

Dietitian, Australia [interview]

Participants described processed foods and additives as pragmatic intervention targets because they could often be addressed without imposing broad restrictions on fruit, vegetables, legumes, or other nutritionally valuable foods.

Salt substitutes, tinned soups, processed meats—they're the usual culprits, and easy wins with little impact on diet quality.

Dietitian, Australia [interview]

I try to focus on avoiding processed foods first and keep it simple.

Dietitian, France [Online free text]

However, participants also recognised that reducing processed foods or additives was not always straightforward. Some patients relied on processed or convenience foods because of cost, time constraints, cooking skills, disability, or food insecurity. In these circumstances, advice needed to be individualised rather than framed as simple avoidance.

I do recommend restricting potassium additives, however there are some cases where they cannot be entirely restricted, for example food insecure populations.

Dietitian, Australia [interview]

The feasibility of detailed additive education also varied by service context. Some participants described limited time, staffing, or follow‐up capacity, which could constrain the extent to which label reading and processed‐food counselling could be explored in routine practice.

In reality, we don't always get the chance to go into processed foods in detail…it's diet recall and ‘avoid X’ for now until we see you in outpatients.

Dietitian, UK [Online free text]

Overall, participants positioned potassium additives and highly processed foods as early and proportionate targets for dietary intervention. This strategy was not presented as universally feasible or sufficient, but as a way of reducing potassium exposure while minimising unnecessary restriction of nutritionally valuable whole foods.

### 3.5 Theme 3: Maintenance of Nutritional Adequacy as a Central Consideration

This theme describes how concerns about nutritional adequacy and dietary quality shaped dietary decision‐making, particularly in relation to energy intake, protein provision, fibre intake, dietary variety, and enjoyment of food. Participants described potassium management as inseparable from broader nutritional risk, especially in patients who were frail, elderly, nutritionally vulnerable, or at risk of protein–energy wasting.

You can't fix potassium at the expense of nutrition…that's not a good trade.

Dietitian, Canada [interview]

Our patients are often already struggling to eat enough. Any restriction must be very carefully balanced.

Dietitian, Sweden [Online free text]

Participants frequently described using the least restrictive approach possible. Rather than defaulting to broad restriction of fruits, vegetables, legumes, or wholegrains, they sought to preserve dietary variety and focus on specific sources of potassium that could be modified without substantially compromising nutritional adequacy.

Overall, I try to be the least restrictive as possible, and I only restrict plant‐based foods as the last resort as we know that higher intakes have better outcomes.

Dietitian, UK [Online free text]

I prefer to identify a few specific high potassium sources, the removal of which won't significantly affect their overall dietary nutritional quality.

Dietitian, Canada [Interview]

In some cases, participants explicitly described prioritising nutritional intake over biochemical targets when the two were in conflict, particularly for patients with frailty, poor appetite, or limited oral intake.

If someone is frail, I will always prioritise intake over restriction.

Dietitian, Australia [Online free text]

This balancing process was described as ongoing rather than fixed, with participants adjusting recommendations in response to changing clinical status, biochemical trends, appetite, and patient priorities.

It's a constant balancing act between the numbers and the person in front of you.

Dietitian, Sweden [Online free text]

Participants also described using whole‐diet approaches to maintain dietary quality while managing potassium exposure. These included encouraging dietary variety, maintaining fibre intake, supporting bowel regularity, and using food preparation strategies, substitutions, or portion modification rather than simple food avoidance.

I generally suggest a ‘plant forward’ diet.

Dietitian, Finland [Online free text]

Plenty of whole grains and fluids to avoid constipation and maintain healthy gut bacteria.

Dietitian, Australia [Interview]

Overall, nutritional adequacy functioned as a key constraint on the extent and intensity of potassium restriction. Dietary strategies were adapted to reduce potassium exposure while maintaining energy intake, protein intake, fibre intake, dietary variety, and quality of life. In this way, participants positioned dietary hyperkalaemia management as part of whole‐person nutrition care rather than as isolated potassium reduction.

### 3.6 Theme 4: Selective Focus on Potassium‐Rich Whole Foods

This theme describes how focusing in on potassium‐rich whole foods, including fruits, vegetables, legumes, and wholegrains, was typically positioned as a later‐stage and conditional intervention with a defined timeline. Participants did not describe whole‐food reduction as an automatic response to hyperkalaemia. Instead, it was framed as a selective strategy used when elevated serum potassium persisted despite consideration of non‐dietary contributors and lower‐burden dietary modifications.

I try everything else first before I start restricting fruits and vegetables. Even Bananas…you can have those as the potassium in them is not 100% absorbable.

Dietitian, UK [online free text]

I avoid banning fruit and veg unless I absolutely must. It's harmful, demoralising, and often unnecessary.

Dietitian, Ireland [online free text]

Where restriction was considered necessary, participants described modifying intake rather than excluding foods outright. Advice was commonly framed around portion size, frequency of consumption, food preparation, and the overall pattern of intake. This reflected an effort to reduce potassium exposure proportionately while preserving dietary variety, fibre intake, cultural acceptability, and enjoyment of food.

We talk about portion size/frequency of food/fluid items… often you do not need to completely remove [potassium]‐rich sources.

Dietitian, Canada [interview]

It's not about banning foods; it's about adjusting how they're eaten and in what quantities. Everyone talks about restrictions and banning foods, and that's what we are up against…this restriction culture. It's difficult to practise in it sometimes. Patients are given it by doctors, nurses, and non‐renal dietitians, and it's a real shame given what we currently know.

Dietitian, UK [Online free text]

Participants also described a “scale‐back” approach, in which potassium intake was reduced from the patient's existing baseline rather than managed through universal lists of prohibited foods. This allowed advice to be individualised according to the patient's usual intake, nutritional status, biochemical trends, and capacity to make dietary changes.

I use a ‘scale‐back’ method where we work on reducing [potassium] intake from baseline levels.

Dietitian, Australia [Interview]

When whole‐food restriction was implemented, it was usually targeted towards concentrated or high‐volume sources rather than single foods eaten occasionally. Participants reported prioritising liquid potassium sources such as juices, smoothies, soups, and tomato‐based sauces, as well as large portions or habitual combinations of higher‐potassium foods which were consumed every day. This approach enabled more precise modification of dietary potassium exposure while avoiding unnecessary restriction of nutritionally valuable foods.

Avoid concentrated sources of potassium such as juice, smoothies, tomato sauces/soups

Dietitian, Netherlands [Online free text]

Participants emphasised that thresholds for initiating whole‐food restriction varied across clinicians and settings, reflecting differences in clinical urgency, perceived risk, local practice norms, patient complexity, and service capacity. Whole‐food restriction was therefore not described as a fixed stage reached by all patients, but as a context‐dependent response to persistent or clinically significant hyperkalaemia.

Dietary advice was also adapted to patients' cultural eating patterns, food availability, food security, food preparation skills, and preferences. This tailoring was described as important for minimising unnecessary dietary burden, maintaining trust, and supporting engagement with dietary advice.

One size does not fit all; advice needs to be patient specific and tailored.

Dietitian, Ireland [Online free text]

Overall, participants positioned restriction of potassium‐rich whole foods as a proportionate and individualised intervention rather than a default rule. Whole‐food restriction was used selectively, modified through portioning or frequency where possible, and continually balanced against nutritional adequacy, quality of life, and the patient's wider clinical context.

### 3.7 Synthesis of Findings and Development of the PRIME‐K Model

Across accounts, dietary management of hyperkalaemia was described as a staged but flexible process of professional reasoning. Participants commonly described prioritising non‐dietary contributors and lower‐burden dietary modifications before considering more restrictive dietary approaches. Dietary modification was therefore not positioned as an automatic response to elevated serum potassium, but as one possible intervention within a broader process of clinical attribution, nutritional assessment, and contextual judgement.

I rarely go straight to restriction unless I've exhausted other options. And then its only if it's absolutely necessary.

Dietitian, UK [Online free text]

However, participants emphasised that this hierarchy was not strictly linear. Clinical urgency, patient complexity, biochemical trends, nutritional risk, patient preferences, and service constraints could alter the sequencing, timing, and combination of interventions. In practice, dietitians described moving between stages while continuing to hold the overall reasoning structure in mind.

In theory it's stepwise, but in practice it's much more fluid. You need to be able to move about but still consider all the steps in your head.

Dietitian, Canada [Interview]

**Figure 1:**
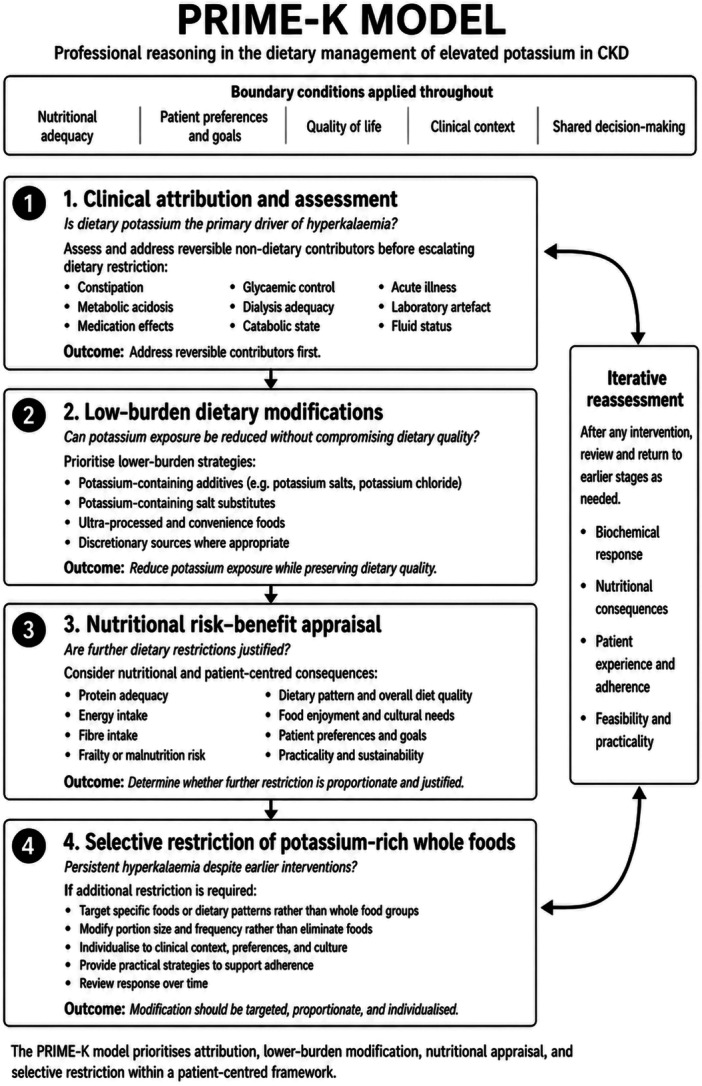
The prioritisation and reasoning in the management of elevated potassium in chronic kidney disease model [The PRIME‐K model].

The findings were synthesised into the Prioritisation and Reasoning in the Management of Elevated Potassium in Chronic Kidney Disease model, known as PRIME‐K (Figure 1). The model represents the reported structure of professional reasoning in dietary hyperkalaemia management and comprises of interrelated components: prioritising clinical attribution before dietary restriction; reducing lower‐burden, high‐bioavailability potassium sources first; individualising advice according to patient context and feasibility; maintaining nutritional adequacy and dietary quality; escalating dietary restriction selectively and proportionately; and keeping potassium response under review.

Decision‐making incorporated biochemical management alongside nutritional adequacy, dietary quality, patient preferences, quality of life, food access, cultural context, and service constraints. The PRIME‐K model therefore makes explicit how renal dietitians reported integrating and prioritising these factors in practice.

The model is intended as a descriptive and conceptual representation of reported practice rather than a prescriptive algorithm. Movement through the model is iterative rather than fixed, with progression, de‐escalation, or simultaneous action shaped by clinical urgency, biochemical trends, nutritional risk, and patients' individual circumstances that shape what dietary advice is realistic, safe, and acceptable for that person.

## 4 Discussion

This study provides a practice‐informed account of how renal dietitians reason through dietary hyperkalaemia management in chronic kidney disease. Rather than describing dietary restriction as an automatic response to elevated serum potassium, participants reported a staged but flexible process of professional reasoning. This involved considering non‐dietary contributors, prioritising lower‐burden dietary modifications such as potassium additives and highly processed foods, protecting nutritional adequacy and dietary quality, and reserving restriction of potassium‐rich whole foods for situations where it was clinically indicated. The PRIME‐K model synthesises these findings into a descriptive representation of how renal dietitians reported prioritising, sequencing, and adapting dietary interventions in routine practice.

The contribution of this study does not lie in identifying entirely new dietary strategies for hyperkalaemia management. Many individual components described by participants, including medication review, constipation management, attention to potassium additives, portion modification, and avoidance of unnecessary fruit and vegetable restriction, are already reflected to varying degrees in contemporary renal nutrition guidance and discourse [6, 7, 8, 9]. The contribution of the present study is to show how these components are integrated through professional reasoning. The data informing these accounts should not be interpreted as evidence of clinical effectiveness. Rather, participants described strategies they perceived as useful for making sense of complex clinical situations, including how they prioritised information, managed uncertainty, and adapted decisions to context. The study was designed to explore dietitians' perspectives and did not assess patient outcomes, diagnostic accuracy, treatment effectiveness, or comparative performance; therefore, the strategies reported here cannot be regarded as clinically proven. They are best understood as practice‐based insights into how renal dietitians reason in context. In this respect, PRIME‐K shifts attention from what dietary strategies are used to how renal dietitians determine when, why, and to what extent those strategies are appropriate.

This distinction is important because dietary hyperkalaemia management has historically been represented through food lists, nutrient thresholds, and restriction‐focused advice. While such approaches may offer pragmatic support for clinicians and patients, they can under‐represent the complexity of potassium homoeostasis and the broader nutritional consequences of dietary restriction. Serum potassium is influenced by dietary intake, medication use, acid–base status, glycaemic control, bowel function, dialysis adequacy, catabolic state, and pre‐analytical factors. Participants' accounts reflected this complexity, with dietary intervention positioned as one part of a wider process of clinical attribution rather than as the default explanation for elevated potassium.

The PRIME‐K model should therefore be interpreted as a conceptual model of reasoning rather than a prescriptive pathway. Its value lies in making explicit the sequencing, proportionality, and boundary‐setting that participants described in practice. Nutritional adequacy and dietary quality functioned not as secondary considerations but as constraints on the intensity of potassium restriction. Similarly, patient context, food access, cultural eating patterns, treatment burden, and service capacity shaped whether and how dietary advice could be implemented. This supports a view of dietary hyperkalaemia management as adaptive professional practice rather than simple adherence to food lists or nutrient thresholds [3].

Participants' accounts also highlighted the role of professional judgement in areas where evidence underpinning practice is uncertain. For example, strategies such as targeting potassium additives, reducing concentrated potassium sources, or modifying whole‐food intake were not presented as universally effective or sufficient. Rather, they were used proportionately, often as pragmatic attempts to reduce plausible potassium exposure while minimising nutritional and psychosocial harm. This is important in an area where serum potassium is influenced by multiple dietary and non‐dietary factors, and where the relationship between individual foods and biochemical outcomes may be difficult to isolate in routine practice.

The prioritisation of potassium additives and highly processed foods is consistent with emerging discussion around potassium bioavailability and nutrient non‐equivalence [3, 9]. Potassium salts used in food processing may be more bioavailable than potassium contained within intact plant‐cell matrices, while whole plant foods also provide fibre, micronutrients, and cardiometabolic benefits. However, participants did not present additive‐focused advice as a complete solution. Instead, it was framed as a proportionate first‐line dietary strategy that could reduce potassium exposure while avoiding unnecessary restriction of nutritionally valuable foods. This distinction is important, as it avoids replacing one simplistic dietary rule e.g., restrict all potassium food additives, with another.

The findings also align with contemporary approaches to chronic kidney disease nutrition that emphasise dietary quality, plant‐forward dietary patterns, fibre intake, cardiometabolic health, and patient‐centred care [6, 7, 8, 9]. Participants frequently described concern that broad restriction of fruits, vegetables, legumes, and wholegrains could undermine these priorities, particularly for patients already at risk of poor intake, frailty, constipation, or protein–energy wasting. Dietary potassium management was therefore understood not simply as correcting a biochemical abnormality, but as one component of broader, person‐centred nutritional care.

Professional reasoning provides a useful lens through which to interpret these findings. Participants' accounts showed that dietetic decision‐making in the management of hyperkalaemia extended beyond the interpretation of biochemical markers or application of dietary guidance. Dietitians described balancing potassium‐related risk with nutritional adequacy, quality of life, food access, cultural acceptability, and patients' capacity to implement dietary change. In this sense, professional reasoning encompassed clinical, ethical, contextual, and relational judgements about what advice was appropriate, feasible, and person‐centred. This aligns with literature on professional judgement in dietetics and other health professions, where dietitians are required to integrate evidence, guidelines, clinical experience, and patient context in complex real‐world settings [14].

These findings may also be understood through the lens of adaptive expertise. Adaptive expertise refers to the capacity to apply underlying principles flexibly, particularly in situations characterised by uncertainty, complexity, or competing priorities [15]. Participants did not describe abandoning structure; rather, they described holding a structured sequence in mind while moving fluidly between assessment, dietary modification, monitoring, and escalation. PRIME‐K captures this combination of structure and flexibility, making visible the cognitive work involved in proportionate dietary decision‐making.

The findings have implications for multidisciplinary communication. Participants described practising within clinical environments where dietary potassium advice may still be communicated in simplified or restrictive terms, such as blanket avoidance of fruits or vegetables. Such messages may be pragmatic in some contexts, particularly where urgent action is required, but they risk reinforcing unnecessary dietary restriction and may undermine dietetic advice aimed at preserving dietary quality. PRIME‐K may provide a structure for articulating renal dietitians' specialist reasoning to other professionals, supporting more consistent and proportionate communication about dietary hyperkalaemia management.

The model may also have value for dietetic education and reflective practice. For students, early‐career dietitians, and non‐specialist clinicians, hyperkalaemia management may appear to involve memorising high‐ and low‐potassium foods. PRIME‐K reframes this task as a reasoning process involving attribution, prioritisation, individualisation, nutritional protection, proportionate escalation, and ongoing review. Used educationally, the model could support case‐based teaching, supervision, and reflective discussion about how dietary decisions are made under conditions of uncertainty.

At a service level, these findings suggest the importance of recognising the cognitive and relational work involved in dietary hyperkalaemia management. Advice to reduce potassium intake may appear straightforward, but participants' accounts showed that proportionate intervention requires time to assess non‐dietary contributors, understand habitual intake, identify feasible changes, negotiate patient preferences, and monitor response. Services that rely on highly standardised or restriction‐focused materials may risk under‐supporting this more nuanced work. Conversely, services that enable dietetic assessment, follow‐up, and multidisciplinary collaboration may be better placed to deliver individualised and nutritionally protective care.

### 4.1 Strengths and Limitations

This study has several strengths. It draws on a large international sample of renal dietitians and integrates survey free‐text responses with semi‐structured interviews, allowing exploration of clinical reasoning across a range of practice contexts. The focus on professional reasoning, rather than isolated dietary practices, provides insight into how dietitians structure and prioritise decision‐making in the management of dietary hyperkalaemia in CKD.

Several limitations should be considered. First, the findings are based on self‐reported accounts of practice and may not fully reflect real‐time clinical decision‐making. Second, the sample may under‐represent dietitians working in non‐English‐speaking contexts, which may limit transferability. Participants' country and work setting were reported to provide context for the dietitian cohort. However, more detailed contextual variables, including rurality or remoteness of practice, local resource availability, and participant diversity, equity and inclusion characteristics, were not collected. This limits interpretation of how these factors may have influenced dietitians' views or practice patterns. Study materials and interviews were conducted in English and were not translated, which may have reduced inclusivity for non‐English‐speaking dietitians. These factors should be considered when assessing the transferability of findings beyond affluent, English‐speaking healthcare contexts. Third, the study primarily reflects the perspectives of experienced renal dietitians and therefore may not capture how clinical reasoning develops in early‐career dietitians. Although the interview sample was small, the qualitative component was designed to supplement and deepen interpretation of the larger survey dataset rather than to generate an independently saturated qualitative account. Small qualitative samples are methodologically acceptable where the purpose is depth and contextual understanding, with Creswell and Poth (2016) noting that samples may involve as few as 3–4 participants and commonly range up to 10 participants [16].

In addition, the model presented reflects synthesis of reported practice rather than evaluation of clinical effectiveness. As a result, it should be interpreted as a descriptive representation of reasoning processes rather than an assessment of clinical outcomes or best practice. Further research is needed to explore how these reasoning processes align with clinical outcomes and evidence‐based practice and how they evolve across levels of professional experience.

### 4.2 Future Research

Further research is needed to examine how these reasoning processes are enacted in real‐time practice. Observational studies, case‐based interviews, think‐aloud methods, or chart‐stimulated recall could explore how dietitians make decisions during clinical encounters and how these decisions are shaped by multidisciplinary input, service constraints, and patient response. Further work could also examine whether PRIME‐K is useful in dietetic education, supervision, guideline implementation, or multidisciplinary communication. Finally, intervention studies are needed to evaluate the clinical and patient‐centred outcomes of commonly used dietary strategies, including additive‐focused advice, reduction of concentrated potassium sources, and proportionate modification of whole‐food intake.

## 5 Conclusion

This study provides insight into how renal dietitians manage dietary hyperkalaemia in CKD, highlighting a staged and adaptive process of clinical reasoning rather than reliance on immediate or uniform dietary restriction. Across accounts, dietitians described prioritising non‐dietary contributors and lower‐burden dietary modifications before considering restriction of potassium‐rich whole foods, with nutritional adequacy acting as a consistent constraint on intervention intensity.

These findings suggest that dietary management of hyperkalaemia is shaped by ongoing balancing of biochemical targets with nutritional risk and patient‐centred considerations. The PRIME‐K model offers a practice‐informed representation of this reasoning process and may support further discussion of how clinical decision‐making is conceptualised in renal nutrition practice.

Further exploration of how these reasoning processes are enacted in real‐time clinical practice, and how they are influenced by organisational context and multidisciplinary decision‐making, would help to extend and test the applicability of these findings beyond self‐reported accounts.

## Author Contributions

A.M. conceptualised and led the study, designed the methodology, oversaw data analysis, and wrote the first draft of the manuscript. K.L. contributed to the development of data collection materials, including refinement of the research questions, undertook data collection, supported data analysis, and contributed to the development and refinement of the PRIME‐K model. Both authors critically reviewed manuscript drafts approved the final version of the manuscript.

## Funding

The authors have nothing to report.

## Conflicts of Interest

The authors declare no conflicts of interest.

## Supporting information


Supporting File

## Data Availability

Data sharing not applicable to this article as no datasets were generated or analysed during the current study.
