# Supplementary material for: Professional Reasoning in Dietary Hyperkalaemia Management in Chronic Kidney Disease: Development of the PRIME‐K Model
Source: J Hum Nutr Diet. 2026 Jul 17;39(4):e70314. doi: 10.1111/jhn.70314 (PMC13377905; doi:10.1111/jhn.70314)
Supplement: Supplementary file 1 — Supporting File [file JHN-39-0-s001.docx]

| **Question Number** | **Interview Question** |
| --- | --- |
| 1 | How do you receive a notification or referral that a patient has hyperkalemia? |
| 2 | How do you prioritise these notifications or referrals? |
| 3 | Can you describe your nutritional assessment process for a patient with hyperkalemia? |
| 4 | How do you feel when you are asked to see a patient with hyperkalemia? |
| 5 | What helps you facilitate good management of hyperkalemia? |
| 6 | Where do you find the joy in your practice around the dietary management of serum potassium? |
| 7 | What do you consider the benefits of referring patients to a dietitian for hyperkalemia management? |
| 8 | What are the barriers you face in the management of hyperkalemia? |
| 9 | What do you find stressful or challenging in your practice around the dietary management of serum potassium? |
| 10 | What would be your top three key messages you would tell your interns or students about hyperkalemia management? |
| 11 | Is there anything else you would like to add about this topic? |

**Table S1 Semi-structured interview questions**
